# C-reactive protein as a predictor of mortality in patients affected with severe sepsis in intensive care unit

**DOI:** 10.1186/2049-6958-7-47

**Published:** 2012-11-21

**Authors:** Özkan Devran, Zuhal Karakurt, Nalan Adıgüzel, Gökay Güngör, Özlem Yazıcıoğlu Moçin, Merih Kalamanoğlu Balcı, Ece Çelik, Cüneyt Saltürk, Huriye Berk Takır, Feyza Kargın, Adnan Yılmaz

**Affiliations:** 1 Department of Pulmonology, Respiratory Intensive Care Unit, SB Süreyyapaşa Chest Diseases and Thoracic Surgery Teaching and Research Hospital, Soyak Yenişehir Manolya Evleri B3/63, Ümraniye, Istanbul, Turkey

**Keywords:** Intensive care unit, Severe sepsis, Serum CRP follow up, SOFA score

## Abstract

**Background:**

Severe sepsis is a primary cause of morbidity and mortality in the intensive care unit (ICU). Numerous biomarkers have been assessed to predict outcome and CRP is widely used. However, the relevance for mortality risk of the CRP level and the day when it is measured have not been well studied. We aimed to assess whether initial and/or third dayCRP values are as good predictors of mortality in ICU patients with severe sepsis as other well-known complex predictors of mortality, i.e., SOFA scores.

**Methods:**

An observational cohort study was performed in a 20-bed respiratory ICU in a chest disease center. Patients with severe sepsis due to respiratory disease were enrolled in the study. SOFA scores**,** CRP values on admission and on the third day of hospital stay, and mortality rate were recorded. ROC curves for SOFA scores and CRP values were calculated.

**Results:**

The study included 314 ICU patients with sepsis admitted between January 2009 and March 2010. The mortality rate was 14.2% (n = 45). The area under the curve (AUC) for CRP values and SOFA scores on admission and on the 3rd day in ICU were calculated as 0.57 (CI: 0.48-0.66); 0.72 (CI: 0.63-0.80); 0.72 (CI: 0.64-0.81); and 0.76 (CI: 0.67-0.86), respectively. Sepsis due to nosocomial infection, a CRP value > 100 mg/L and higher SOFA scores on 3rd day, were found to be risk factors for mortality (odds ratio [OR]: 3.76, confidence interval [CI]: 1.68-8.40, p < 0.001, OR: 2.70, CI: 1.41-2.01, p < 0.013, and OR: 1.68, CI: 1.41-2.01, p < 0.0001, respectively).

**Conclusions:**

The risk of sepsis related mortality appears to be increased when the 3rd day CRP value is greater than 100 mg/dL. Thus, CRP appears to be as valuable a predictor of mortality as the SOFA score.

## Background

For the last decade we have known that, to decrease ICU mortality, identifying sepsis and quickly implementing its therapyis crucial. The ‘surviving sepsis campaign’ [1] has been implemented in emergency and intensive care units (ICU) all over the world, with significant results in sepsis therapy. In the case of patients with sepsis, APACHE II [2] is used to predict mortality, and the sepsis related organ failure assessment (SOFA) [3] is used to evaluate the response to therapy. However, many parameters are required for these scores, and **it** is not always practical in the clinical setting. A cheap, they are rapid, easy method to evaluate therapy response and predict mortality of patients with severe sepsis requiring ICU treatment is needed. There are a limited number of studies that describe baseline C-reactive protein (CRP) (an inflammatory marker) level, and its changes in response to therapy, as a predictor of sepsis mortality. Although one study claims that assessing serum CRP levels is not an adequate test for predicting sepsis related to mortality in ICU patients [4], other studies indicate that CRP is a proper predictor of mortality [5,6]. Recently, ∆ CRP was shown to be a predictor of mortality from sepsis in dogs [7]. We aimed to assess whether in patients with severe sepsis requiring admission to ICU the initial and/or third day CRP values could be as good predictors of mortality as other well known complex predictors of mortality (i.e., SOFA scores, and APACHE II).

## Methods

This study had the local hospital approval of the Internal Review Board. The study was conducted in a 20-bed respiratory ICU in a chest disease center of a tertiary teaching hospital. The ICU is a non-surgical, non-obstetrical, respiratory unit. Pulmonologists run the ICU and a pulmonologist is available 24 hours a day. All patients had acute respiratory failure and the majority of patients had chronic obstructive pulmonary disease (COPD).

### Patients

Between January 1st, 2009 and March 31st, 2010 814 patients with respiratory failure admitted to the ICU were assessed. Out of these, 314 fulfilled the sepsis criteria [8] for enrollment into the study; they had been in the ICU for more than three days, and serum CRP had been assessed. Exclusion criteria were a diagnosis of cancer, or rheumatic disease possibly causing a high CRP level. In all cases the reasons for sepsis were related to respiratory diseases such as acute exacerbation of COPD, bronchiectasis, TB sequelae, and pneumonia.

### Modified protocol for surviving sepsis

The *Early Directed Goal Therapy (EDGT)* protocol was followed as described [1]. *Moderate tidal volume*[9]; the protocol was based on providing a tidal volume not greater than 6 mL/kg per ideal body weight. *Moderate dose steroids*[10]: Stress-dose steroid therapy was given only in cases of septic shock after blood pressure was identified to be poorly responsive to fluid and vasopressor therapy (basal cortisol or ACTH stimulation tests were not obtained as they were not available in our hospital). Due to the absence of hydrocortisone in our country, methyl prednisolone was used at a dose of 20 mg tid for 7 days [10] in patients without contra-indications. *Glucose Control* protocol [11]; if blood glucose was > 150 mg/dL, a continuous intravenous insulin infusion was titrated to maintain blood glucose levels between 110 and 140 mg/dL (< 150 mg/dL).

### Mechanical ventilation

In our unit, if the patient was alert, able to protect the airway, had no risk of aspiration, and no burn or wound on the face, non-invasive mechanical ventilation (NIMV) was the first choice of ventilatory support for hypercapnic and hypoxemic respiratory failure [12]. Invasive mechanical ventilation (IMV) was applied with ICU ventilators (Puritan Bennett 760, Newport and Vela) if the patient had criteria for intubation such as cardiac arrest, increased work of breathing, respiratory depression, shock, and altered mental status. Assist control ventilation (A/C), either pressure control or volume control, was preferred as the initial ventilation mode. In volume control ventilation, inspiratory flow was set to provide an airway plateau pressure < 35 cmH2O, and tidal volume was managed as 6–8 mL/kg ideal body weight. During mechanical ventilation a sedation protocol was applied. The Richmond agitation sedation (RAS) scale was used for infusion and assessment of the daily need for sedation [13].

### Laboratory records

The complete blood count (CBC), serum biochemistry, and CRP levels of patients were recorded on the first day in ICU. CBC and blood electrolytes were checked every day and CRP was checked on the 3rd day of ICU (control). The SOFA score was calculated on the 1st and 3rd day in ICU, and APACHE II was calculated on the initial and the discharge day. Initial arterial blood gases (ABGs) were recorded. Serum CRP was checked by the nephelometry method with a BN ProSpecT machine (DADE Behring). The normal range of CRP is 0–5 mg/L. Initial and control CRP levels were checked and ∆ CRP was calculated by subtracting control CRP from the initial value.

### Microbiology

Bronchial secretions of patients were collected by deep tracheal aspiration into the tracheal aspirate tube (if the patient was intubated). In non-intubated patients sputum was collected into a sputum Petri dish. In the case of a very low or high fever (<36°C or >38°C) a blood sample was collected into an aerobic culture media.

### Statistical analysis

This was a prospective clinical study concerning prognosis. The clinical features, ABGs, SIRS criteria, SOFA score on the first and third day, initial, control, and ∆ CRP levels, and comorbidities of survivors and non-survivors were compared. The Mann–Whitney *U* test and Student’s *t* test were used for analysis of continuous variables with non-parametric and parametric values, respectively. The chi square test was applied for categorical variables (gender, comorbidity, status of IMV and NIMV) of survivors and non-survivors. The logistical regression model was used for baseline, control, and ∆ CRP levels to predict the relation to mortality. Other parameters such as nosocomial infection, APACHE II and SOFA score on the first and third day (believed to reflect hospital mortality) were also added to the logistical regression model. The area under the curve (AUC) was used to compare initial and third day SOFA scores, basal, control, and ∆ CRP levels.

## Results

Of the 314 patients included in this study, 208 (66.2%) were male, and the median age was 64 years (interquartile range (IQR) 51–72). The age and SIRS criteria of the patients are summarized in Table 1.

**Table 1:** Results of all patients’ characteristics, ICU data and the comparison of survivors and nonsurvivors

	**All patients (N. 314)**	**Survivors (N. 269)**	**Non-survivors (N. 45)**	p*
	**Median (IRQ,25%-%75)**	**Median (IRQ,25%-%75)**	**Median (IRQ,25%-%75)**	
Age	64 (51–72)	64 (51–72)	64 (47–71)	0.54
Gender n F/M	106/208	92/177	14/31	0.69
Hospital acquired sepsis %	21.9	18.2	44.4	0.000
***ICU severity scores& CRP***				
APACHE II 1st day	19 (15–24)	18 (15–23)	23 (20–28)	0.000
SOFA score 1st day	4 (3–6)	4 (3–5)	7 (4–9)	0.000
SOFA score 3rd day	3 (2–4)	3 (2–4)	6 (3–8)	0.000
CRP mg/L initial	69.0 (26.9-136.0)	67.2 (26.0 133.0)	91.3 (35.2-161.0)	0.12
CRP mg/L after 3–5 day in ICU	50.9 (18.4-97.5)	44.3 (17.9-88.5)	105.0 (61.0-159.0)	0.000
APACHE II 1st day	16.8 (−12.2; 55.1)	18.3 (−6.2;55.1)	−1.7 (−42.3;53.0)	0.040
***Arterial blood gas values***				
pH values	7.32 (7.25- 7.42)	7.34 (7.26-7.42)	7.27 (71.8-7.42)	0.017
PaCO_2_ mmHg	66.0 (44.4-82.0)	65.8 (44.9-80.0)	66.6 (43.5-84.4)	0.81
PaO_2_ mmHg	69.9 (54.8-92.7)	70.6 (55.0-92.0)	65.8 (53.8-97.0)	0.62
Sat O_2_ %	92.9 (85.4-96.4)	93.0 (87.0-96.3)	90.0 (81.9-96.0)	0.17
PaO_2_/FiO_2_	169.9 (117.8-235.3)	173.0 (123.3-236.3)	142.0 (97.5-225.0)	0.041
***Hospital & ICU days***				
Pre ICU hospital days	3 (2–8)	3 (1–7)	4 (2–9)	0.39
Length of stay in ICU	8 (5–13)	8 (5–12)	9 (6–15)	0.19
Length of stay in hospital	15 (11–21)	15 (11–21)	15 (9–21)	0.48
***Mechanical ventilation***				
Days of IMV,	6 (3–11)	6 (3–11)	7 (4–13)	0.10
IMV, % (N)	36.6 (115)	31.2 (84)	68.9 (31)	0.000
Days of NIV	6 (4–10)	6 (4–10)	5 (4–9)	0.41
NIV, % (N)	82.2 (258)	85.5 (230)	62.2 (28)	0.000

*ICU*: Intensive care unit, *CRP*: C- reactive protein (N: 0–5 mg/L), *APACHE II*: acute physiologic and chronic health evaluation II, *SOFA*: Sepsis related Organ Failure Assessment, *PaCO*_*2*_: partial pressure of carbon dioxide, *PaO*_*2*_: partial pressure of oxygen, *SaO*_*2*_: arterial oxygen saturation, *FIO*_*2*_: Fraction of inspired oxygen, *IMV*: invasive mechanical ventilation, *NIMV*:noninvasive mechanical ventilation. *p values for comparing the survivors and nonsurvivors data, Mann–Whitney Test.

**Figure 1:**
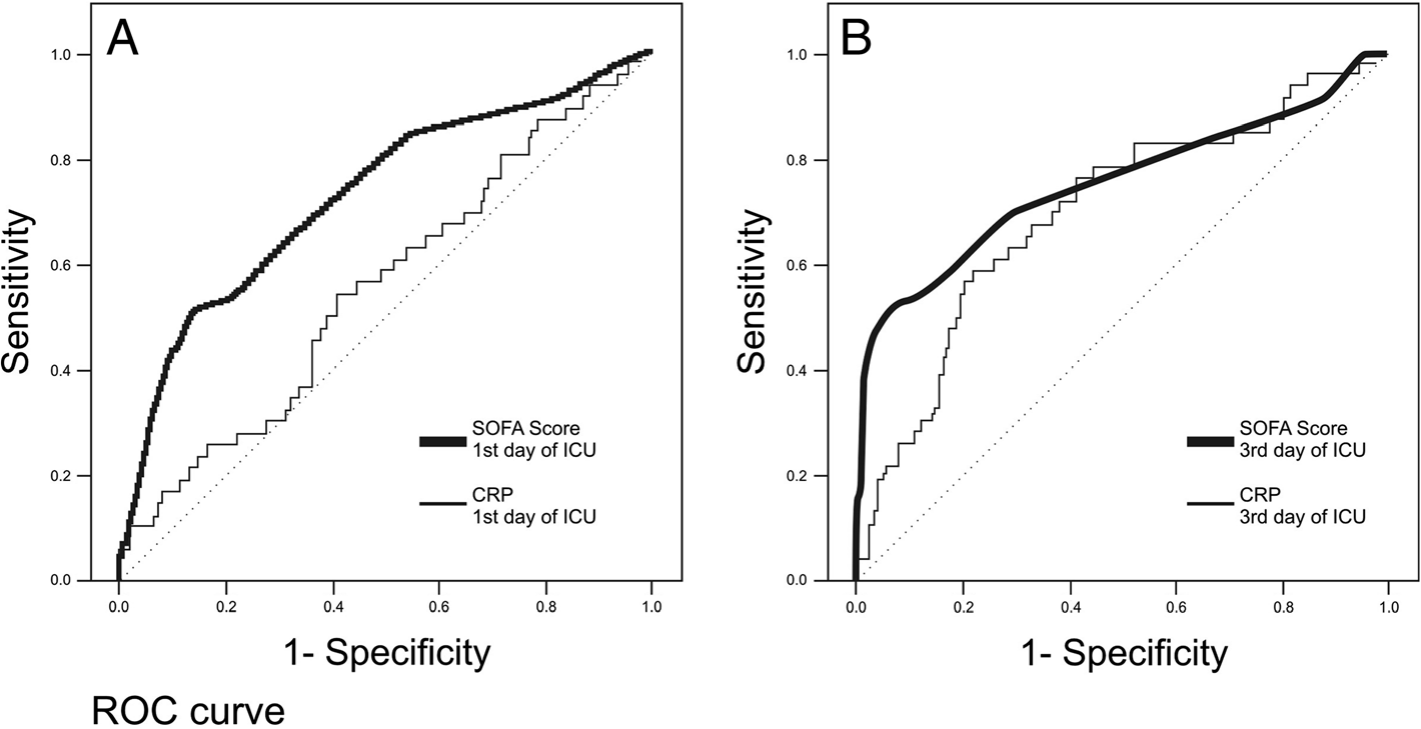
**On the left side, the ROC curve for mortality of the first day of ICU, CRP and SOFA scores were shown.** On the right side, the ROC curves for mortality on the third day, CRP and SOFA scores were drawn.

Initial APACHE II and SOFA scores, CRP levels, SOFA scores on the third day, ∆ CRP and CRP levels on the 3rd day are shown in Table 1. The ABG values of patients on admission to ICU are also summarized in Table 1. The majority of patients had COPD and their ABG values demonstrated acute or chronic hypercapnia with hypoxemia. The location from which enrolled patients were admitted were the emergency room (118 [37.6%]), inpatient clinic (162 [51.6%]), and other ICUs (34 [10.8%]). In-hospital days on ICU admission, hospital and ICU length of stay (LOS), days of mechanical ventilation for invasive (n = 115, 36.6%) and non-invasive (n = 258, 82.2%) mechanical ventilation are shown in Table 1. Hospital acquired sepsis was present in 69 cases (21.9%) at the time of ICU admission. For 161 patients (51.3%) diagnostic procedures such as bronchial lavage, deep tracheal aspiration, blood, and urine cultures were performed to identify the microorganism causing sepsis. Agents were identified by culture positivity in 63 cases (20.1%) and *P. aeruginosa* was the major pathogen isolated (n = 29, 9.2%). The ICU mortality was 14.2% (n = 45). The clinical features, means of mechanical ventilation, CRP, APACHE II, and SOFA scores of survivors and non-survivors are compared in Table 1. The relation between SOFA scores on the first and third days, baseline and 3rd day of ICU CRP, and ∆ CRP values and mortality were used to draw ROC curves. For the SOFA score on the first day AUC: 0.72 ± 0.04, CI: 0.64-0.81, p < 0.0001; SOFA score on the third day AUC: 0.76 ± 0.05, CI: 0.67-0.86, p < 0.0001; ICU admission CRP AUC: 0.57 ± 0.05, CI: 0.48-0.66, p > 0.117; CRP on the 3rd day AUC: 0.72 ± 0.04, CI: 0.63-0.80, p < 0.0001; and ∆ CRP AUC: 0.41 ± 0.05, CI: 0.30-0.50, p < 0.040. Figure 1 shows the ROC curves on the day of ICU admission and on the third day of ICU for CRP and SOFA scores. The third day CRP value was a better predictor of mortality when compared with the first day CRP.

Sepsis related with nosocomial infection, IMV application and the duration thereof, PaO2/FiO2, APACHE II on admission to ICU, SOFA scores (on admission to ICU and on the 3rd day), CRP values (initial, 3rd day, and ∆ CRP) were used to form a multi-logistical regression model. In this model the third day SOFA score, nosocomial sepsis, and the third day CRP > 100mg/L were determined to be risk factors for mortality (Table 2).

**Table 2:** Mortality risk factors for our patients with sepsis

	**p**	**OR**	**95.0% CI**
Nosocomial infection	0.001	3.76	1.68-8.40
SOFA score on 3rd day	0.000	1.68	1.41-2.01
CRP > 100mg/L on 3rd day	0.013	2.70	1.23-15.91

*OR*: Odds ratio; *CI*: confidence intervals; *SOFA scores*: Sepsis related organ failure assessment, *CRP*: C- reactive protein.

## Discussion

In this study we have shown that control CRP levels can be used to predict the mortality of patients with respiratory failure due to sepsis and being treated by the sepsis protocol, as well as the initial APACHE II, and SOFA scores on the first and third day of ICU (well-known predictors of mortality).

Sepsis protocols proposed by the Sepsis Campaign have been successfully used and predicted mortality for sepsis patients with an initial ICU APACHE II was 32.2%, whereas actual mortality was 14.2%. It has been suggested that CRP levels could impact on the prognosis of ICU patients by some studies done in septic dogs and humans [5-7,14]. Gebhardt et al. [7] declared that the change in initial and third day control CRP levels was better for predicting mortality than initial CRP, and a fall in the third day CRP level demonstrated corrected survival with 94% accuracy in a study with septic dogs. In our study, we showed that initial and ∆ CRP levels were not as valuable for predicting ICU mortality in sepsis patients and 3rd day control CRP was better than initial CRP values and ∆ CRP values, respectively (3rd day CRP AUC: 0.72, CI: 0.63-0.80; initial CRP AUC: 0.57, CI: 0.48-0.66; ∆ CRP AUC: 0.41, CI: 0.30-0.50). These results differed from the study on dogs by Gebhardt et al. [7]. Silvestre et al. [4] recently studied the prognostic value of initial APACHE II, SAPS II, SOFA, CRP, fever, and leukocyte count in 158 sepsis patients. The AUC (CI 95%: lower-upper limit) was 0.75 (0.67-0.83), 0.82 (0.75-0.89), 0.80 (0.72-0.88), 0.55 (0.45-0.65), 0.48 (0.38-0.58), and 0.46 (0.35-0.56), respectively. They concluded that CRP was not an adequate test for the prognosis of sepsis patients. Initial CRP was not a good mortality predictor in our study, but control CRP was found to be as significant as the SOFA score for predicting response to sepsis treatment and prognosis. In another study which looked at the relationship between CRP levels and mortality in sepsis patients within the first 24 hours after discharge from ICU, the mean CRP for non-survivors and survivors was 174 mg/L and 85.6 mg/L, respectively. A high CRP level was indicated to be an independent risk factor of mortality [15]. In our study, the CRP level at the time of discharge from the ICU was not recorded. In addition, we did not follow up on hospital mortality after ICU discharge. However, in the present study, after the 3rd-5th day of treatment, the median CRP values were higher in non-survivors than survivors (105 mg/L versus 44 mg/L, respectively). Instead of initial CRP values, CRP values measured a few days after admission may be more helpful for physicians to make judgments on treatment response and sepsis outcome in the ICU. Pro-calcitonin has also been highlighted as an indicator of inflammation due to infection and there are studies indicating the serum levels of pro-calcitonin may be a better indicator of sepsis severity than CRP [16,17]. Pro-calcitonin is much more expensive than serum CRP and we did not use it in this study for financial reasons. It has been noted that using pro-calcitonin in sepsis management could increase the validity of the clinical decision since it yields results rapidly and it has a shorter half-life than CRP (by 19 hours) [5].

SOFA scores on admission to ICU and on the third day were found to be good predictors of mortality risk (AUC for SOFA on the 1st day, 0.72 [CI: 0.64-0.81], SOFA on the 3rd day, 0.76 [CI: 0.67-0.86]). SOFA score is not as practical or as rapid a test as CRP to evaluate sepsis severity as it is calculated by adding platelet count, PaO_2_/FiO_2_ ratio, serum bilirubin, creatinine, and the Glasgow Coma Scale. In our study the 3rd day SOFA score and the 3rd day CRP value were shown to be risk indicators for sepsis related mortality when comparing severity scores. We believe that the 3rd day CRP value can be used in clinical practice in the ICU to reveal mortality risk and is comparable with the SOFA score when its level is high on third day after initiation of treatment for sepsis (> 100 mg/L).

Thus, in this study analyzing risk factors for overall mortality in sepsis and looking for parameters that influence mortality (indicated as significant in bivariate analysis, with logistical regression analysis) a higher SOFA score on the third day, hospital acquired sepsis, and a 3rd day CRP > 100mg/L were shown to be risk factors for mortality. The culture positivity for detecting an infectious agent in hospital acquired sepsis was only 20%. *Pseudomonas* spp was the most common agent in the culture results. The low culture positivity was assumed to be due to ongoing antibioticotherapy at the time of culture and the initiation of empirical therapy before collection of the culture specimen. In a previous study where CRP levels were checked every four days in critical patients with a 38% rate of culture positivity it was concluded that a ≥ 50 mg/L fall in CRP levels could be a good predictor of recovery [18]. In our study there was a median fall of 18.3 mg/L in ∆ CRP in survivors.

## Conclusions

Sepsis is a preventable pathology. Instead of looking at high CRP levels on the day of ICU admission due to sepsis, a CRP level > 100 mg/L on the third day of ICU may be as good a predictor of mortality as a high SOFA score.

## Competing interests

There isn’t any financial research support and conflict of interest for this study. All authors have not any disclosure.

## References

[B1] Dellinger RP Carlet JM Masur H Gerlach H Calandra T Cohen J Gea-Banacloche J Keh D Marshall JC Parker MM Ramsay G Zimmerman JL Vincent JL Levy MM Surviving Sepsis Campaign Management Guidelines Committee: Surviving Sepsis Campaign guidelines for management of severe sepsis and septic shock Crit Care Med 2004 32 858 873 10.1097/01.CCM.0000117317.18092.E4 15090974

[B2] Knaus WA Draper EA Wagner DP Zimmerman JE APACHE II: A severity of disease classification system Crit Care Med 1985 13 818 829 10.1097/00003246-198510000-00009 3928249

[B3] Vincent JL Moreno R Takala J Willatts S De Mendonça A Bruining H Reinhart CK Suter PM Thijs LG The SOFA (Sepsis-related Organ Failure Assessment) score to describe organ dysfunction/failure. On behalf of the Working Group on Sepsis-Related Problems of the European Society of Intensive Care Medicine Intensive Care Med 1996 22 707 710 10.1007/BF01709751 8844239

[B4] Silvestre J Póvoa P Coelho L Almeida E Moreira P Fernandes A Mealha R Sabino H Is C-reactive protein a good prognostic marker in septic patients? Intensive Care Med 2009 35 909 913 10.1007/s00134-009-1402-y 19169668

[B5] Lobo SM Lobo FR Bota DP Lopes-Ferreira F Soliman HM Mélot C Vincent JL C-reactive protein levels correlate with mortality and organ failure in critically ill patients Chest 2003 123 2043 2049 10.1378/chest.123.6.2043 12796187

[B6] Prieto MF Kilstein J Bagilet D Pezzotto SM C-reactive protein as a marker of mortality in intensive care unit Med Intensiva 2008 32 424 430 10.1016/S0210-5691(08)75719-X 19080865

[B7] Gebhardt C Hirschberger J Rau S Arndt G Krainer K Schweigert FJ Brunnberg L Kaspers B Kohn B Use of C-reactive protein to predict outcome in dogs with systemic inflammatory response syndrome or sepsis J Vet Emerg Crit Care (San Antonio) 2009 19 450 458 10.1111/j.1476-4431.2009.00462.x 19821886

[B8] Bone RC Balk RA Cerra FB Dellinger RP Fein AM Knaus WA Schein RM Sibbald WJ Definitions for sepsis and organ failure and guidelines for the use of innovative therapies in sepsis. The ACCP/SCCM Consensus Conference Committee. American College of Chest Physicians/Society of Critical Care Med Chest 1992 101 1644 1655 10.1378/chest.101.6.1644 1303622

[B9] The Acute Respiratory Distress Syndrome Network Ventilation with lower tidal volumes as compared with traditional tidal volumes for acute lung injury and the acute respiratory distress syndrome N Engl J Med 2000 342 1301 1308 10793162 10.1056/NEJM200005043421801

[B10] Briegel J Forst H Haller M Schelling G Kilger E Kuprat G Hemmer B Hummel T Lenhart A Heyduck M Stoll C Peter K Stress doses of hydrocortisone reverse hyperdynamic septic shock: a prospective, randomized, double-blind, single-center study Crit Care Med 1999 27 723 732 10.1097/00003246-199904000-00025 10321661

[B11] Finfer S Chittock DR Su SY Blair D Foster D Dhingra V Bellomo R Cook D Dodek P Henderson WR Hébert PC Heritier S Heyland DK McArthur C McDonald E Mitchell I Myburgh JA Norton R Potter J Robinson BG Ronco JJ NICE-SUGAR Study Investigators Intensive versus conventional glucose control in critically ill patients N Engl J Med 2009 360 1283 1289 19318384 10.1056/NEJMoa0810625

[B12] Majid A Hill NS Noninvasive ventilation for acute respiratory failure Curr Opin Crit Care 2005 11 77 81 10.1097/00075198-200502000-00012 15659949

[B13] Sessler CN Gosnell MS Grap MJ Brophy GM O'Neal PV Keane KA Tesoro EP Elswick RK The Richmond Agitation-Sedation Scale: validity and reliability in adult intensive care unit patients Am J Respir Crit Care Med 2002 166 1338 1344 10.1164/rccm.2107138 12421743

[B14] Seller-Pérez G Herrera-Gutiérrez ME Lebrón-Gallardo M de Toro-Peinado I Martín-Hita L Porras-Ballesteros JA Serum C-reactive protein as a marker of outcome and infection in critical care patients Med Clin (Barc) 2005 125 761 765 10.1016/S0025-7753(05)72184-9 16373024

[B15] Ho KM Lee KY Dobb GJ Webb SA C-reactive protein concentration as a predictor of in-hospital mortality after ICU discharge: a prospective cohort study Intensive Care Med 2008 34 481 487 10.1007/s00134-007-0928-0 17992507

[B16] Mitaka C Clinical laboratory differentiation of infectious versus non-infectious systemic inflammatory response syndrome Clin Chim Acta 2005 351 17 29 10.1016/j.cccn.2004.08.018 15563869

[B17] Meng FS Su L Tang YQ Wen Q Liu YS Liu ZF Serum procalcitonin at the time of admission to the ICU as a predictor of short-term mortality Clin Biochem 2009 42 1025 1031 10.1016/j.clinbiochem.2009.03.012 19324026

[B18] Reny JL Vuagnat A Ract C Benoit MO Fagon JY Diagnosis and follow-up of infections in intensive care patients: value of C-reactive protein compared with other clinical and biological variables Crit Care Med 2002 30 529 535 10.1097/00003246-200203000-00006 11990910

